# Implementing sodium–glucose co-transporter 2 inhibitors in cardiovascular–kidney–metabolic syndrome: a multidisciplinary expert perspective

**DOI:** 10.1093/ehjcvp/pvag020

**Published:** 2026-04-10

**Authors:** Angelo Avogaro, Mauro Gori, Giuseppe Grandaliano, Massimo Iacoviello, Roberto Trevisan

**Affiliations:** Laboratory of Experimental Diabetology, Veneto Institute of Molecular Medicine, Via Giuseppe Orus 2, 35129 Padua, Italy; Division of Cardiology, Cardiovascular Department, Papa Giovanni XXIII Hospital, 24127 Bergamo, Italy; Department of Translational Medicine and Surgery, Università Cattolica del Sacro Cuore, 00168 Rome, Italy; Nephrology, Dialysis and Transplantation Unit, Fondazione Policlinico Universitario A. Gemelli IRCCS, 00168 Rome, Italy; Department of Medical and Surgical Sciences, University of Foggia, 71122 Foggia, Italy; Endocrinology and Diabetes Unit, Azienda Ospedaliera Papa Giovanni XXIII, 24127 Bergamo, Italy; Department of Medicine and Surgery, University of Milano Bicocca, 20126 Milan, Italy

**Keywords:** Cardio–kidney–metabolic syndrome, Cardio–renal–metabolic syndrome, Cardiovascular–kidney–metabolic syndrome, CKM syndrome, Cardiovascular, renal, and metabolic disorders, SGLT2 inhibitors, Sodium-glucose co-transporter 2 inhibitors

## Abstract

**Aims:**

Cardiovascular–kidney–metabolic (CKM) syndrome defines a pathophysiological continuum driven by reciprocal dysfunction across the cardiovascular, renal and metabolic systems. Although sodium–glucose co-transporter 2 inhibitors (SGLT2i) provide consistent, organ-protective benefits across this spectrum, clinical implementation remains suboptimal. This expert opinion, developed by a multidisciplinary Italian board, aims to translate current evidence and guideline recommendations into practical, integrated strategies for the early and effective implementation of SGLT2i in patients at risk of or affected by CKM syndrome.

**Methods and results:**

The panel reviewed the latest clinical trial data, international guidelines, and real-world evidence to identify implementation gaps and propose actionable solutions across diabetology, cardiology, and nephrology. Clinical recommendations were formulated via informal multidisciplinary roundtable discussions. Despite strong evidence and broad guideline endorsement, SGLT2i remain underutilized due to fragmented care, therapeutic inertia, and misconceptions regarding safety. To address these barriers, we advocate for early risk-based screening, simplified treatment algorithms, cross-specialty collaboration, and educational efforts to empower both clinicians and patients.

**Conclusion:**

Shifting from reactive to proactive CKM management requires an integrated care model aligning specialties around early, organ-protective interventions. SGLT2i should be recognized as foundational, disease-modifying therapy, supported by multidisciplinary coordination, clear clinical algorithms, and patient-centered communication.

## Introduction

Cardiovascular–kidney–metabolic (CKM) syndrome embodies a complex and dynamic interaction among the cardiovascular, renal, and metabolic systems. Rather than a simple coexistence of comorbidities, CKM represents a progressive pathophysiological continuum in which impairment in one system accelerates deterioration in the others, thereby affecting clinical outcomes.^1–5^ With the global rise of type 2 diabetes (T2D), chronic kidney disease (CKD), and heart failure (HF), CKM has become a key challenge for healthcare professionals.

Managing CKM requires an integrated, holistic and evidence-based approach that encompasses specialty boundaries and enables early, coordinated interventions. This perspective builds upon prior expert consensus advocating a transition from reactive, event-driven care to a proactive, syndromic, risk-stratified model.^2^ This expert opinion extends this trajectory by addressing practical challenges and outlining cross-specialty strategies to integrate emerging therapies into routine clinical workflows. Among these, sodium–glucose co-transporter 2 inhibitors (SGLT2i)—the first pharmacologic class to consistently demonstrate cardio-renal-metabolic benefits across multiple large-scale trials, including non-diabetic populations—now occupy a central role in CKM management.^6–14^

Leading international guidelines, including those from the American Heart Association (AHA), American Diabetes Association (ADA), Kidney Disease: Improving Global Outcomes (KDIGO), European Society of Cardiology (ESC) and European Society of Hypertension (ESH), now recommend SGLT2i for organ protection in patients at risk of cardiovascular and renal complications, irrespective of glycaemic control.^3,15–23^ Yet, despite this compelling evidence, real-world uptake of SGLT2i remains inconsistent, hindered by delayed diagnosis, fragmented care pathways, therapeutic inertia, and ambiguity regarding prescribing responsibilities.

To address these gaps, a multidisciplinary advisory board of Italian experts in cardiology, nephrology, and diabetology convened in June 2025. During the meeting, experts discussed current CKM care practices, key barriers to early SGLT2i use, and practical strategies to improve cross-specialty integration; no formal consensus methodology was applied. A non-systematic search was conducted on PubMed related on CKM syndrome and the clinical implementation of SGLT2i, focusing on high-impact publications, large-scale randomized controlled trials, and real-world evidence registries published between 2015 and 2026.

The recommendations presented in this expert opinion reflect the synthesis of available evidence and expert discussion, with the aim of translating guideline recommendations into actionable, practice-oriented strategies for integrated CKM care.

## Current recommendations on sodium–glucose co-transporter 2 inhibitors in cardiovascular–kidney–metabolic syndrome

The growing body of clinical evidence has firmly established SGLT2i as a cornerstone therapy across the CKM spectrum. The benefits of this class of drugs extend well beyond glycaemic control: robust data indicate a significant risk reduction of HF hospitalizations, CKD progression, and major cardiovascular events (MACE). These outcomes support the repositioning of SGLT2i as disease-modifying agents with multi-organ protective effects.^7–14^ Mechanistic studies suggest anti-inflammatory and antioxidant actions that disrupt the vicious cycle of tissue injury and chronic inflammation inherent to CKM progression.^11,12^

This strong evidence has led the major medical societies to incorporate SGLT2i into guidelines for cardiovascular, renal, and metabolic diseases. This section summarizes the key recommendations from diabetology, nephrology, and cardiology guidelines, supporting a unified approach to the patient with CKM risk.^3,4,14–23^ A comprehensive overview of these indications across clinical scenarios is provided in *Table 1*.

- The ADA Standards of Care recommend SGLT2i for adults with T2D and either established or high risk of HF, CKD, or atherosclerotic cardiovascular disease, regardless of glycaemic status or metformin use.^15–17^
- The KDIGO Clinical Practice Guideline strongly endorses SGLT2i in patients with T2D and CKD with an estimated glomerular filtration rate (eGFR) ≥20 mL/min/1.73 m^2^, particularly if HF or albuminuria is present (urinary albumin-to-creatinine ratio—uACR ≥200 mg/g), to prevent kidney and cardiovascular events.^18^
- The ESC Heart Failure guidelines recommend SGLT2i for all patients with HF (both reduced and preserved ejection fraction), independent of diabetes status, to reduce hospitalization and cardiovascular death.^19,20^
- The ESC Guidelines on Chronic Coronary Syndromes (CCS) and the AHA/ACC Chronic Coronary Disease Guidelines recommend SGLT2i for T2D patients with established atherosclerotic cardiovascular disease or CCS to reduce MACE. Additionally, SGLT2i are recommended for patients with CCS and reduced left ventricular ejection fraction (LVEF) (≤40%) regardless of T2D status.^21,22^
- The ESH Guidelines for the management of arterial hypertension recommend SGLT2i to prevent HF in T2D patients with hypertension, and for blood pressure (BP) management in HF patients.^23^

**Table 1:** Summary of guideline indications for sodium–glucose co-transporter 2 inhibitors in key cardiovascular–kidney–metabolic phenotypes^16–23^

Patient clinical presentation	Key clinical criteria	Class/level of recommendation	Guideline recommending SGLT2i
T2D with cardiovascular/renal risk	T2D + CVD or HF or CKD or high CV risk	1A	ADA Standards of Care 2025 + ESC 2023 + ESH 2023 + KDIGO Diabetes & CKD Guideline
CKD with or without T2D	eGFR ≥20 mL/min/1.73 m^2^	1A	KDIGO Diabetes & CKD Guideline + ADA 2025 CKD
	eGFR ≥20 mL/min/1.73 m^2^ and uACR^a^ ≥200 mg/g or HF	1A	KDIGO Diabetes & CKD Guideline
	eGFR 20–45 mL/min/1.73 m^2^ and uACR^a^ <200 mg/g	2B	KDIGO Diabetes & CKD Guideline
HF (HFrEF or HFpEF) with or without T2D	Established HF (HFrEF or HFpEF) or high-risk	1A	ESC HF Guideline Update 2023 + ADA 2025
ASCVD with T2D	T2D + indicators of established ASCVD (CCS, PAD, history of MI/stroke) or high CV risk	1A	ESC HF Guideline Update 2023 + ADA 2025 + 2024 ESC Guidelines for the management of CCS + 2023 AHA/ACC
CCS with T2D	T2D + CCS (stable coronary artery disease, prior MI, revascularization) or established ASCVD	1A	2024 ESC Guidelines for the management of CCS + 2023 AHA/ACC
CCS with or without T2D	CCS + HF with LVEF^a^ ≤40%	1A	2023 AHA/ACC + 2023 ESC HF
Hypertension or HF with T2D	T2D + hypertension, HF or BP needs	1A	ESH Hypertension Guidelines 2023 + ESC 2023

AHA/ACC, American Heart Association/American College of Cardiology; ASCVD, atherosclerotic cardiovascular disease; BP, blood pressure; CCS, chronic coronary syndrome; CKD, chronic kidney disease; CVD, cardiovascular disease; eGFR, estimated glomerular filtration rate; HFpEF, heart failure with preserved ejection fraction; HFrEF, heart failure with reduced ejection fraction; LVEF, left ventricular ejection fraction; MI, myocardial infarction; PAD, peripheral artery disease; T2D, type 2 diabetes; uACR, urine albumin-to-creatinine ratio.

^a^Estimated glomerular filtration rate values are expressed as mL/min/1.73 m^2^ and correspond to the eGFR used in KDIGO CKD staging. Albuminuria thresholds are expressed as uACR; a value ≥200 mg/g indicates moderate-to-severe albuminuria according to KDIGO criteria. LVEF ≤40% defines HF with reduced ejection fraction (HFrEF) according to ESC and AHA/ACC heart failure guidelines.

**Figure 1:**
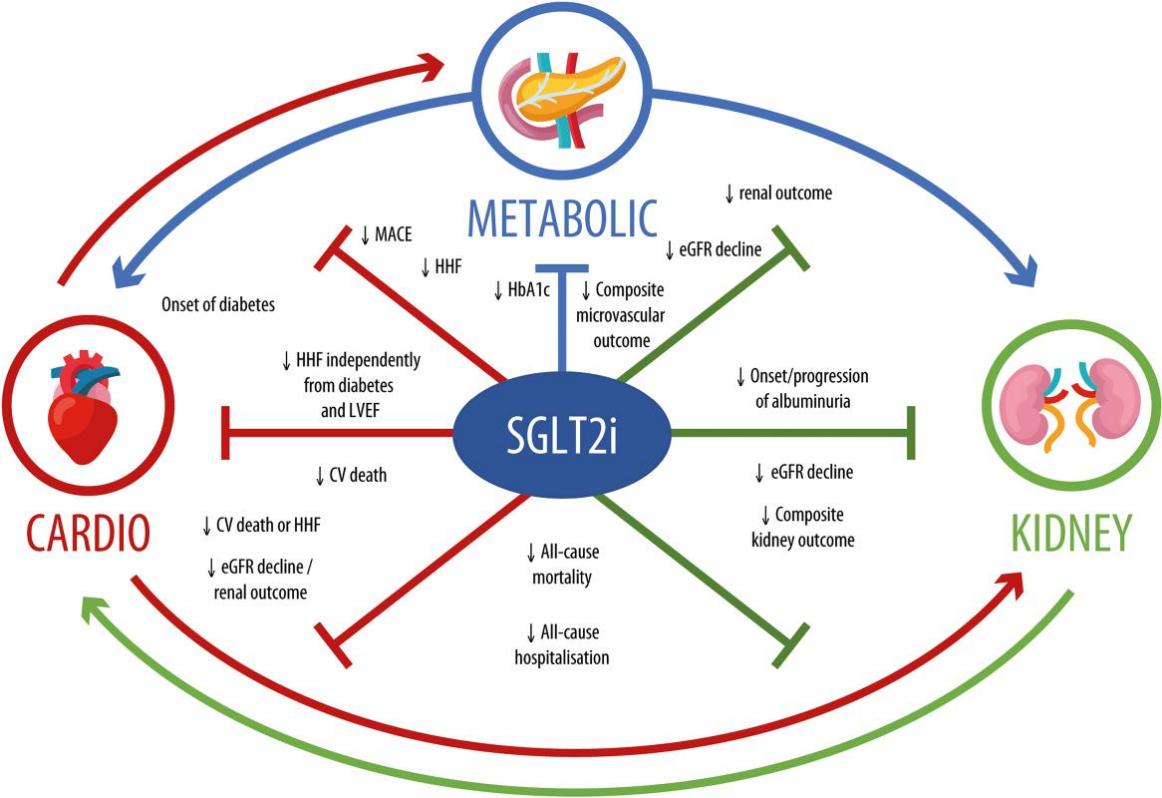
Organ-protective effects of sodium–glucose co-transporter 2 inhibitors in cardiovascular–kidney–metabolic syndrome continuum. Sodium–glucose co-transporter 2 inhibitors exerts beneficial effects across the cardiovascular–kidney–metabolic continuum. They reduce the risk of major cardiovascular events, hospitalization for heart failure, progression of chronic kidney disease, and improve metabolic control, underscoring their systemic, disease-modifying potential. CV, cardiovascular; eGFR, estimated glomerular filtration rate; HbA1c, haemoglobin A1c; HHF, hospitalization for heart failure; LVEF, left ventricular ejection fraction; MACE, major adverse cardiovascular events; SGLT2i, sodium–glucose cotransporter-2 inhibitors (Original).

Together, these statements underscore a major paradigm shift: SGLT2i are not simply glucose-lowering drugs but organ-protective therapies that cross traditional specialty boundaries. This integrated efficacy is visually represented in *Figure 1*, which illustrates the multifaceted benefits of SGLT2i throughout the CKM continuum.

The convergence of recent guidelines underscores the need for a holistic, integrated approach to CKM care. Instead of managing cardiovascular, renal, and metabolic diseases in isolation, clinicians should apply unified, risk-based algorithms that enable early intervention—well before irreversible organ damage develops.

Coordinated screening strategies, incorporating eGFR, albuminuria, and cardiac biomarkers, are crucial for identifying eligible patients across specialties. Equally vital is conveying to patients that SGLT2i are not simply glucose-lowering drugs but agents that provide broad, multi-organ protection, an understanding that enhances adherence and fosters alignment across specialties.

## Clinical challenges and barriers to the use of sodium–glucose co-transporter 2 inhibitors in daily practice

Despite robust evidence and guideline endorsement, the real-world implementation of SGLT2i in clinical practice remains suboptimal. Persistent clinical, operational, and systemic barriers reflect broader issues of fragmentation, uncertainty, and therapeutic inertia across specialties.^2,3,10,12^ Data from national and international real-world registries consistently reveal substantial implementation gaps, with many eligible patients, particularly those with T2D, HF or CKD, not properly treated with SGLT2i. Importantly, therapeutic inertia is not unique to SGLT2i. Historical data across cardiovascular and renal care have consistently shown underuse and suboptimal dose optimization of renin–angiotensin–aldosterone system (RAAS) inhibitors, despite their well-established prognostic benefit in HF, CKD, and high cardiovascular risk populations.^3,23^ Therefore, the current implementation gap observed with SGLT2i should be interpreted within a broader systemic challenge affecting multiple components of guideline-directed medical therapy (GDMT), rather than as a class-specific phenomenon.

### Evidence from real-world evidence registries

In the diabetology field, the Annals from Italian *Associazione Medici Diabetologi* (AMD) 2024 registry (over 1.5 million patients) reports that only 41.9% of T2D individuals receive SGLT2i.^24^ Among high-risk subgroups, over half of those with eGFR <60 mL/min and one-third with albuminuria remain untreated, as do 32.6% with HF and 26.3% with prior cardiovascular events.

In cardiology, the BRING-UP 3 registry highlights persistent variability in SGLT2i uptake across heart failure phenotypes.^25^ Usage reached 84.4% in HF with reduced ejection fraction (HFrEF) but dropped to 72.1% in HF with Mildly Reduced Ejection Fraction (HFmrEF) and 50.1% in HF with preserved ejection fraction (HFpEF). Barriers included renal dysfunction and previous genitourinary infections (69%), alongside operational issues such as delayed initiation or limited access to lab testing (31%). Similarly, SWEDEHEART registry data show that in T2D patients hospitalized for acute myocardial infarction, fewer than 5% were discharged on SGLT2i between 2012 and 2017,^26^ rising only to 22.2% by 2021.^27^ Older age, female sex, and reduced eGFR were the main limiting factors.^26,27^ The TITRATE-HF registry reported particularly low SGLT2i use in *de novo* HF (3%), compared with 64% in chronic and 56% in worsening HF.^28^ In HFrEF, only 65.4% received SGLT2i.^28^

In nephrology, the CURE-CKD registry (over 39 000 patients with T2D and CKD) found only 5.5% received SGLT2i at baseline, with poor persistence.^29^ Similarly, the CKD-REAL study noted delayed initiation and frequent discontinuation, mainly due to concerns about eGFR decline or genitourinary infections, despite overall good tolerability.^30^

*Table 2* provides a synthesis of these findings across clinical settings. These data collectively confirm that underuse of SGLT2i spans all specialties. While the reasons vary, from clinical misperceptions to structural inefficiencies, the consequences are missed opportunities to prevent progression of CKM and reduce cardio-renal risk.

**Table 2:** Summary of RWE registries on sodium–glucose co-transporter 2 inhibitors use

Clinical setting	Registry (with patient details)	Key findings on SGLT2i use	Barriers to prescription
Diabetology	Annali AMD 2024^24^ Over 1.5 million T2D patients	41.9% of T2D patients on SGLT2i; 54% of T2D with eGFR <60^a^ and 33% with albuminuria^a^ not treated with SGLT2i; 32.6% with HF and 26.3% with prior CV events not on SGLT2i/GLP-1 RA	Lack of prescription despite high cardio-renal risk; implementation gap not attributable to low risk
Cardiology	BRING-UP 3^25^ 3830 HF outpatients	SGLT2i use: 84.4% in HFrEF^a^, 72.1% in HFmrEF^a^, 50.1% in HFpEF^a^; 15.7% of HFrEF not treated with SGLT2i	69% of non-prescriptions due to clinical barriers (renal dysfunction, infections); 31% due to operational issues
	SWEDEHEART (RIKS-HIA)^26,27^ T2D patients post-AMI (2012–21)	<5% of eligible post-AMI T2D patients treated with SGLT2i (2012–17); increased to 22.2% at discharge (2018–21)	Older age, female sex, low eGFR; renal function <30^a^ as main exclusion
	TITRATE-HF^28^ 4288 patients with *de novo*, chronic, or worsening HF	SGLT2i at baseline: 3% in *de novo* HF^a^, 64% in chronic HF, 56% in worsening HF; 65.4% in HFrEF	81% of omissions undocumented; 19% due to contraindications (mainly genitourinary infections)
Nephrology	CURE-CKD^29^ 39 179 patients with diabetes and CKD	6% received SGLT2i at baseline; only 5.0% maintained SGLT2i ≥90 days; ACEi/ARB 70.7%; GLP-1 RA 6.8%	Lower use in uninsured or Medicaid patients; women less likely to receive or persist; hospitalization associated with discontinuation
	CKD-REAL^30^ 271 patients with Stage 4 or non-dialysis Stage 5 CKD	Initiation delayed by median 356 days from diagnosis; 10.3% discontinued within 6 months	Discontinuation mainly due to eGFR decline or AKI (69.2%), followed by genitourinary infections (10.3%); inertia in response to initial eGFR changes

ACEi, angiotensin-converting enzyme inhibitors; AKI, acute kidney injury; AMI, acute myocardial infarction; ARB, angiotensin II receptor blockers; CKD, chronic kidney disease; CV, cardiovascular; eGFR, estimated glomerular filtration rate; GLP-1 RA, glucagon-like peptide-1 receptor agonists; HF, heart failure; HFmrEF, heart failure with mildly reduced ejection fraction; HFpEF, heart failure with preserved ejection fraction; HFrEF, heart failure with reduced ejection fraction; KDIGO, Kidney Disease: Improving Global Outcomes; SGLT2i, sodium–glucose cotransporter-2 inhibitors; T2D, type 2 diabetes.

^a^Estimated glomerular filtration rate values are expressed as mL/min/1.73 m^2^ and refer to the eGFR used to define CKD severity. Albuminuria thresholds refer to uACR; values ≥30 mg/g indicate clinically relevant albuminuria according to KDIGO classification. HF phenotypes are defined according to ESC guidelines: HFrEF (LVEF ≤40%), HFmrEF (LVEF 41%–49%), and HFpEF (LVEF ≥50%).

Beyond clinical factors, these registries highlight that real-world implementation of GDMT is heavily influenced by healthcare systems and reimbursement policies. In the CURE-CKD registry, high out-of-pocket costs and local formulary restrictions remain critical barriers, with uninsured or non-commercially insured patients significantly less likely to receive SGLT2i or glucagon-like peptide-1 receptor agonists (GLP-1 RAs).^29^ Conversely, in many European healthcare systems with broader public reimbursement frameworks, direct patient costs may be less prominent barriers; however, implementation can still be influenced by national prescribing regulations, specialist-initiated therapy requirements, or local formulary restrictions.^25,28^ From a health-system perspective, however, growing evidence indicates that early use of SGLT2i is cost-effective due to reductions in HF hospitalizations and slowing of CKD progression.^13,18^

### Beyond the numbers: clinical and organizational barriers

Beyond simple numerical underuse, multiple interrelated barriers continue to limit the routine uptake of SGLT2i, including clinical hesitation, fragmented care pathways, and organizational variability, many of which echo those documented in contemporary registry analyses.

A central issue is the absence of a unified framework for early identification and initiation. Diabetologists, cardiologists, and nephrologists often encounter early signs of CKM risk, yet fragmented protocols and siloed guidelines prevent coordinated action. As a result, patients with ‘single-domain’ conditions, such as isolated T2D or CKD, often miss timely, risk-based treatment. All clinicians, regardless of specialty, should be empowered to recognize early warning signs and act before multisystem damage occurs.

Prescribing hesitation also arises from perceived patient complexity. Concerns regarding polypharmacy, frailty, hypotension, and comorbidities often delay initiation. While several safety concerns reflect misconceptions, others represent legitimate clinical considerations that require appropriate monitoring rather than therapeutic avoidance. The transient eGFR drop that follows SGLT2i initiation is a reversible and well-characterized haemodynamic effect associated with long-term nephroprotection, rather than structural kidney injury. Similarly, increased haematocrit indicates a beneficial marker, yet often mistaken for an adverse signal. These misconceptions, reflected in registry-based discontinuations, perpetuate inertia despite strong safety data.^31^

Genitourinary infections, commonly cited adverse events, are typically mild and self-limiting, and do not require permanent discontinuation. Most patients can safely resume treatment after resolution, especially if supported by education and preventive measures;^32^ however, they remain a leading cause of early discontinuation. Basic hygiene advice and reassurance can markedly improve adherence and should be systematically integrated into treatment initiation pathways.^32^

However, clinicians should recognize that SGLT2i may increase the risk of volume depletion and postural hypotension, particularly in lean or older individuals, those with advanced HF, or patients receiving concomitant diuretics or antihypertensive therapy.^13^ This perception reflects the limited representation of low-body mass index (BMI) populations in major trials and underscores the need for individualized therapeutic decisions rather than uniform protocol-driven implementation. In such contexts, monitoring of BP, volume status, and potential adjustment of background diuretic therapy may be appropriate rather than deferring treatment.^13^ Euglycaemic diabetic ketoacidosis (DKA), although uncommon in randomized trials, represents another important complication, mainly occurring in patients with T2D and specific precipitating factors such as acute illness, reduced caloric intake, perioperative fasting, or insulin dose reduction.^13^ Structured patient education, sick-day rules, and temporary treatment interruption during high-risk situations are effective strategies to mitigate this risk. To support clinicians in managing these and other safety considerations, *Table 3* provides a practical and operational framework for SGLT2i initiation, monitoring, and perioperative management.

**Table 3:** Practical safety and monitoring framework for sodium–glucose co-transporter 2 inhibitors therapy

Clinical domain	Safety and management protocol
Baseline labs and monitoring	Evaluate a full baseline screening panel including eGFR, uACR, HbA1c, lipid profile, BP, and ECG prior to initiation
	Assess volume status, nutritional status, and DKA risk before starting therapy.^13,17^
	Reassess renal function ∼2–4 weeks after SGLT2i initiation and periodically thereafter according to CKD stage and clinical context.^18^
	Standard periodic monitoring is sufficient unless volume depletion is suspected.^13,17^
Discontinuation thresholds and misconceptions	An initial, reversible eGFR drop is expected and safe.^18,31^
	Clinicians must overcome misconceptions regarding this initial eGFR decline or expected haematocrit increases, which do not indicate toxicity.
	Do not alter therapy for expected early eGFR reductions.^13,18,31^
	Investigate other causes only if the acute eGFR dip is >30% from baseline; even a dip >30% rarely requires permanent SGLT2i discontinuation.^13,18,31^
“Sick day” and surgery rules	Withhold SGLT2i during acute dehydrating illness or vomiting to prevent DKA and AKI.^13,18,33^
	Pause therapy 3–4 days before major surgery or prolonged fasting.^13,18,33^
	Document a clear plan to restart the drug once the patient is stable, euvolemic, and eating normally.^13,18,33^
Drug–drug interactions	Lean or older individuals, and those with advanced HF or concomitant diuretics/antihypertensives, require closer monitoring for postural hypotension and volume depletion.^13^
	Reduce loop/thiazide diuretics if the patient is at risk of hypovolemia; consider reducing sulfonylureas or insulin doses to prevent hypoglycaemia.^13,33^
	SGLT2is are safe to combine with RAASi/MRAs and may even lower the risk of hyperkalaemia.^13,18^
Common side effects	Genital infections are usually mild and occur early in treatment.^13,18,31,33^
	Mitigate risk by providing hygiene counselling and early symptom recognition at treatment initiation.^13,31^
	Treat with standard antifungals; drug discontinuation is rarely needed.^13^

ADA, American Diabetes Association; AKI, acute kidney injury; BP, blood pressure; CKD, chronic kidney disease; DKA, diabetic ketoacidosis; ECG, electrocardiogram; eGFR, estimated glomerular filtration rate; HbA1c, haemoglobin A1c; HF, heart failure; KDIGO, Kidney Disease: Improving Global Outcomes; MRAs, mineralocorticoid receptor antagonists; NICE, National Institute for Health and Care Excellence; RAASi, renin–angiotensin–aldosterone system inhibitors; SGLT2i, sodium–glucose cotransporter-2 inhibitors; uACR, urine albumin-to-creatinine ratio.

A persistent barrier is the absence of integrated guidance across specialties. Without shared CKM frameworks, clinicians may hesitate to prescribe outside their domain of expertise. A shared minimal screening panel, including eGFR and albuminuria, enables early detection and timely intervention. Measurement of N-terminal pro-B-type natriuretic peptide (NT-proBNP) may also be considered in patients presenting with symptoms suggestive of HF. Yet, operational variability, especially in primary care and hospital records, limits this approach.

Although the AHA CKM staging model provides a valuable conceptual framework, it is often perceived as overly linear and cardiocentric. In practice, CKM progression is far more dynamic and bidirectional, with substantial overlaps in pathophysiology across organ systems. As highlighted by Iacoviello *et al*.^2^ a more inclusive, multispecialty model with the diverse clinical entry points across disciplines better captures real-world CKM syndrome complexity.

Finally, the lack of defined responsibility for CKM care undermines continuity and delays intervention. No single specialty is accountable for global management, reinforcing silos and hindering implementation of organ-protective therapies like SGLT2i.^31^ This fragmentation may also affect other cardioprotective therapies, including RAAS inhibitors and mineralocorticoid receptor antagonists (MRA), further emphasizing that improving CKM outcomes requires system-level alignment of multidisciplinary care rather than the promotion of a single pharmacological class.

*Table 4* summarizes the most frequent clinical misconceptions and obstacles that hinder SGLT2i adoption, along with practical, multidisciplinary strategies to overcome them. These challenges underscore the need for shared clinical algorithms, multidisciplinary communication, and a system-wide shift towards proactive care to align clinical practice with evidence and ensure timely and appropriate treatment for patients at risk of CKM.

**Table 4:** Clinical barriers to sodium–glucose co-transporter 2 inhibitors use and practical recommendations

Barrier/misconception	Clinical impact on patient care	Practical recommendations
Transient eGFR decline	Misinterpreted as acute injury, leading to therapy discontinuation or avoidance	Educate clinicians on the haemodynamic mechanism of the transient eGFR dip; monitor but reassure on reversibility
Genitourinary infections	Frequent cause of early discontinuation despite mild nature.	Provide hygiene education and early symptom recognition; emphasize manageability.
Haematocrit increase	Perceived as adverse rather than a marker of haemoconcentration and improved renal perfusion	Clarify prognostic benefit; reinforce that rise is an expected therapeutic effect.
Risk of volume depletion, hypotension, or euglycaemic DKA	Prescribing delay in older, frail, or complex patients	Identify high-risk contexts (advanced HF, acute illness, perioperative fasting); monitor BP and volume status; provide sick-day guidance
Complex patient profiles (frailty, polypharmacy, comorbidities)	Overcautious prescribing due to fear of interactions.	Promote individualized risk–benefit evaluation; simplify multidisciplinary protocols.
Lack of integrated CKM screening	Missed opportunities for early intervention.	Implement ‘8 Life’s Essentials’ and shared screening panels (eGFR, uACR, HbA1c, NT-proBNP, blood pressure, lipids)
Delayed or limited access to laboratory testing	Hinder eligibility confirmation and timely initiation.	Ensure access to baseline labs and implement fast-track initiation protocols
Absence of shared follow-up pathways	Loss of treatment continuity; poor monitoring and early discontinuation	Develop coordinated, multidisciplinary follow-up models with clear communication between specialists and GPs

BP, blood pressure; CKM, cardio-kidney-metabolic; DKA, diabetic ketoacidosis; eGFR, estimated glomerular filtration rate; GP, general practitioner; HbA1c, haemoglobin A1c; NT-proBNP, N-terminal pro–B-type natriuretic peptide; SGLT2i, sodium–glucose cotransporter-2 inhibitors; uACR, urine albumin-to-creatinine ratio.

## Towards an integrated model of cardiovascular–kidney–metabolic care

Overcoming therapeutic inertia and fragmentation in CKM care requires more than awareness; it calls for structural reforms. The barriers outlined in the previous section highlight the urgent need for a proactive, multidisciplinary, and risk-driven approach, rather than reactive disease-specific responses.

**Figure 2:**
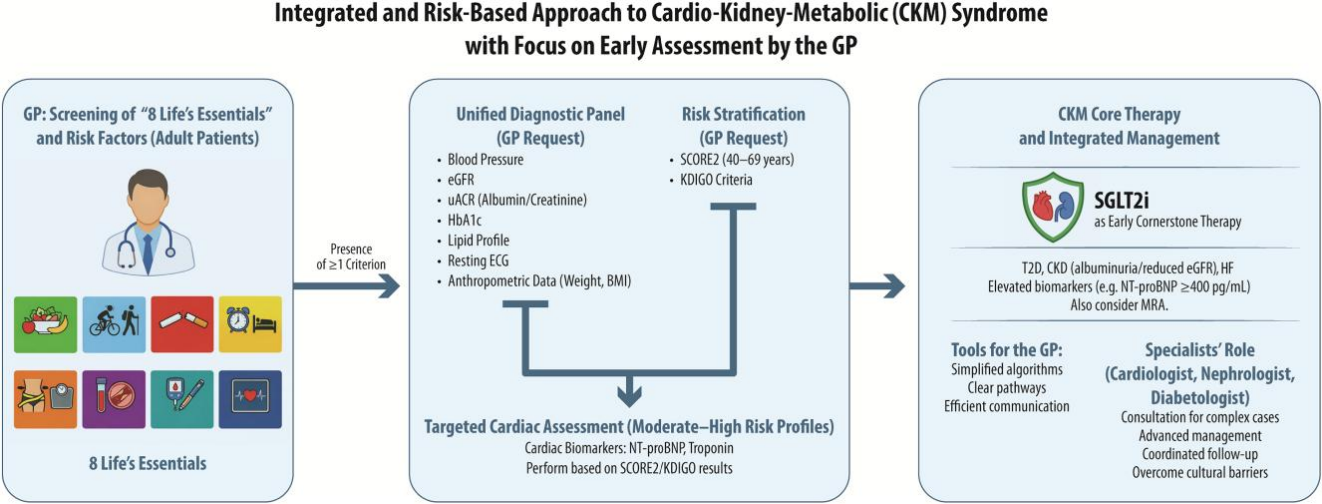
Integrated clinical pathway for the early assessment and management of cardiovascular–kidney–metabolic syndrome. This model centers on the general practitioner for early detection, with initial screening based on the ‘8 Life’s Essentials’. It integrates a stepwise progression from risk identification to a unified diagnostic panel and stratification (incorporating SCORE2 and KDIGO criteria), followed by targeted cardiac biomarker assessment in at-risk profiles. This risk-based approach guides the early initiation of core pharmacotherapies (e.g. sodium–glucose co-transporter 2 inhibitors, mineralocorticoid receptor antagonists) and establishes a collaborative framework between primary care and specialists. CKD, chronic kidney disease; eGFR, estimated glomerular filtration rate; GP, general practitioner; HbA1c, haemoglobin A1c; HF, heart failure; KDIGO, Kidney Disease: Improving Global Outcomes; MRA, mineralocorticoid receptor antagonist; NT-proBNP, N-terminal pro-B-type natriuretic peptide; SCORE2, Systematic Coronary Risk Evaluation 2; SGLT2i, sodium–glucose cotransporter-2 inhibitors; T2D, type 2 diabetes; uACR, urinary albumin-to-creatinine ratio (Original).

The core of this shift is the early identification and stratification of CKM risk. Here, we propose that the ‘8 Life’s Essentials’, BP, glycaemia, body weight, dyslipidaemia, smoking status, physical activity, sleep quality, and diet, could support lifestyle-based prevention from the earliest stages (*Figure 2*), and should become standard in routine evaluations across specialties. A unified diagnostic panel is recommended for all care settings, including BP, eGFR, uACR, HbA1c (haemoglobin A1c), lipid profile, resting electrocardiogram (ECG), and anthropometric data (weight, BMI). Cardiovascular risk assessment using Systematic Coronary Risk Evaluation 2 (SCORE2) is advised in individuals aged 40–69 years, with the addition of NT-proBNP and troponin when needed, such as in selected high-risk profiles. Parallel renal stratification using KDIGO criteria is essential to guide early nephroprotective strategies.

Importantly, this risk-stratified approach should also drive therapy selection. Patients with diabetes, albuminuria, reduced eGFR, or elevated biomarkers warrant consideration for early SGLT2i intervention, even in the absence of overt events. To this end, real-world data from the Italian DARWIN-Renal study confirmed that initiating an SGLT2i effectively prevents kidney function decline and reduces albuminuria even in uncomplicated T2D patients at low baseline renal risk, highlighting its critical value in primary prevention.^34^ In these phenotypes, randomized outcome trials and contemporary guidelines provided direct evidence supporting organ protection.^16–20^ In addition, recent real-world data showed that, in community-based patients with suspected HF, elevated NT-proBNP (≥400 pg/mL) and a pre-existing indication for SGLT2i (e.g. T2D, CKD or resistant hypertension), early initiation of SGLT2i and/or MRA was associated with a substantial reduction in the risk of HF hospitalization or HF events.^35,36^ To support practical implementation across different clinical entry points, an actionable framework for SGLT2i initiation and management, integrating guideline thresholds, phenotypic stratification, and monitoring recommendations, is summarized in *Table 5*.

**Table 5:** Actionable clinical framework for sodium–glucose co-transporter 2 inhibitors initiation and management in cardiovascular–kidney–metabolic syndrome

Phenotype	Initiation criteria and guideline class	Concomitant therapies	Monitoring and stop rules
CKD with T2DM	eGFR: ≥20 mL/min/1.73 m^2^ (1A).^16–18^	RAASi: Recommend ACEi/ARB if uACR ≥30 mg/g.^16–18^ MRAs: Add finerenone if eGFR ≥25 mL/min/1.73 m^2^ and uACR ≥30 mg/g to reduce CKD progression and CV events.^16–18^	Monitoring: Initial eGFR drop (≤ 30%) is expected and safe Stop: Suspend SGLT2i/RAASi during acute dehydrating illness to prevent AKI.
CKD without T2DM	eGFR: ≥20 mL/min/1.73 m^2^ and uACR ≥200 mg/g (1A).^18^ eGFR 20–45 mL/min/1.73 m^2^ and uACR <200 mg/g (1B).^18^	RAASi: Recommend ACEi/ARB if uACR >300 mg/g or consider if uACR 30–300 mg/g.^18^	Monitoring: Initial eGFR drop (≤ 30%) is expected and safe SGLT2i lowers the risk of AKI and fluid overload across all ranges.^18^
HF (HFrEF, HFmrEF, HFpEF)	Symptomatic HF (any LVEF) irrespective of diabetes status.^19–21^ eGFR: ≥20 mL/min/1.73 m^2^ (1A).^17–21^ NT-proBNP ≥400 pg/mL and a pre-existing indication for SGLT2i (e.g. T2D and/or CKD).^35^	Assess volume status; reduce diuretics if hypovolemic risk exists.^13,17^	Monitoring: Routine renal function and volume status Stop: Suspend SGLT2i/diuretics if severe dehydration occurs to avoid hypovolemia.^13,18,23^
T2D (high CV risk/ASCVD)	Foundational for high CV risk/ASCVD, independent of HbA1c. eGFR: ≥20 mL/min/1.73 m^2^ (1A).^15–17^ uACR: Independent of albuminuria ranges.^15–17^	Combine with GLP-1 RA for additive benefits if BMI ≥35, high CV risk, or established ASCVD.^3,16,33^	Monitoring: Educate on genital hygiene to prevent mycotic infections Stop: Suspend 3–4 days prior to elective surgery/fasting.^13,15–17,33^
Advanced age and frailty	No strict age limit. Avoid SGLT2i only if high risk of falls or severe hypotension.^33^	Review polypharmacy and adjust antihypertensives	High risk of volume depletion. Strict ‘sick day’ education is mandatory.^13,33^

ACEi, angiotensin-converting enzyme inhibitors; ARB, angiotensin II receptor blockers; ASCVD, atherosclerotic cardiovascular disease; CKD, chronic kidney disease; CV, cardiovascular; eGFR, estimated glomerular filtration rate; HF, heart failure; HFpEF, heart failure with preserved ejection fraction; HFmrEF, heart failure with mildly reduced ejection fraction; HFrEF, heart failure with reduced ejection fraction; LVEF, left ventricular ejection fraction; MRA, mineralocorticoid receptor antagonist; RAASi, renin–angiotensin–aldosterone system inhibitors; SGLT2i, sodium–glucose cotransporter-2 inhibitors; T2DM, type 2 diabetes mellitus; uACR, urinary albumin-to-creatinine ratio.

The clinical impact of these interventions is supported by robust quantitative data across the CKM spectrum. Recent data in community-based patients with suspected HF indicates that the number needed to treat over 12 months to prevent one event of HF hospitalization or death is 19 for SGLT2i alone, dropping to 12 with SGLT2i + MRA combination therapy, and as low as 7 in very high-risk profiles.^35^ For renal protection, pivotal trials like DAPA-CKD and EMPA-KIDNEY reported absolute risk reductions of 5.3% and 3.8% for their primary kidney-protective endpoints.^1^ From a population perspective, treating 1000 patients with SGLT2i for 3 years prevented up to 16 MACE and 28 HF hospitalizations.^33^ These metrics are consistent with meta-analyses showing that SGLT2i use prevents 34.0 CV deaths or HF hospitalizations per 1000 patient-years in HF patients and 11.0 renal progression events per 1000 patient-years in those with CKD.^13^

This evidence reinforces that SGLT2i must be integrated into a comprehensive GDMT framework alongside RAAS inhibitors. Historically, implementation gaps and therapeutic inertia have hindered the optimal use of RAASi as well, thus the underutilization is a systemic issue in CKM management rather than a class-specific phenomenon.^13^ In this context, SGLT2i should be viewed as complementary to RAASi, as their combined use provides additive cardiorenal benefits and may even mitigate common clinical barriers, such as the risk of hyperkalaemia often associated with RAASi and MRA therapy.^13^ Furthermore, a rapid sequential or parallel initiation of these foundational therapies is now advocated to alter the disease trajectory before irreversible organ damage develops.^13,35,36^

Within this integrated model, SGLT2i and GLP-1 RAs are seen as complementary rather than competing options. A large collaborative meta-analysis (SMART-C) recently confirmed that the cardioprotective and nephroprotective benefits of SGLT2i are highly consistent and safe, irrespective of background GLP-1 RAs use, with no evidence of altered safety profiles when these classes are combined.^37^ Regarding the optimal sequencing of these therapies, a recent observational study demonstrated that starting with an SGLT2i prior to adding a GLP-1 receptor agonist confers significantly greater long-term preservation of eGFR compared with the reverse sequence, reinforcing SGLT2i as a foundational step for kidney protection.^38^ Sodium–glucose co-transporter 2 inhibitors remains the first choice for cardio–renal protection, particularly in HF and CKD, independently of the presence of diabetes, while GLP-1 RAs offer added value in reducing atherosclerotic risk and enhancing metabolic control in patients with diabetes. In parallel, RAAS inhibitors remain central for BP control, reduction of intraglomerular hypertension, and mitigation of neurohormonal activation, reinforcing the need for an integrated, phenotype-driven therapeutic strategy. In selected high-risk individuals, combined use of RAAS inhibition, SGLT2i, and, when appropriate, GLP-1 RA may provide complementary protection across the CKM spectrum.^1,3,5,13^  *Table 6* provides a concise comparative overview of the main CKM disease-modifying therapies, highlighting their principal domains of benefit and their positioning within integrated care pathways.

**Table 6:** Disease-modifying pharmacotherapies across the cardiovascular–kidney–metabolic spectrum: clinical positioning and key considerations

Therapy class	Core CKM domain(s)	Phenotypes where benefit is established	Practical positioning within integrated CKM care	Key clinical considerations
SGLT2i	CV + Renal + Metabolic	HF across all ejection fractions (HFrEF, HFmrEF, HFpEF); CKD with or without T2D; T2D with ASCVD or high CV risk.^1,3,16,18–22,35,36^	Foundational therapy across CKM spectrum and core GDMT pillar for HF. Early initiation is strongly recommended.^3,13,36^	Monitor for volume depletion, genital infections, and rare euglycaemic DKA. Initial eGFR drop (3–10%) is expected and safe.^13,16–18^
GLP-1 RA	CV + Renal + Metabolic	T2D with ASCVD/high CV risk, obesity/overweight with comorbidities; T2DM with CKD; obesity with HEpEF.^1,3,13,16,17,21,22,37,38^	Prioritized for weight loss, severe hyperglycaemia, and ASCVD risk reduction. Often combined with SGLT2i for synergistic benefits.^3,13,16,17,21,22,37,38^	Gastrointestinal side effects; requires withhold during severe illness (sick day rules) or pregnancy/breastfeeding.^1,13,16–18,33^
RAASi (ACEi/ARB)	CV + Renal	Hypertension, CKD (diabetic and non-diabetic) with albuminuria; HFrEF.^3,16–23,25^	First-line for hypertension with CKD or proteinuria, for managing HFrEF and slowing CKD.^3,16–23^	An initial eGFR drop (≤ 30%) is expected (Mutruc 2025). Monitor for hyperkalaemia and uraemia, especially in advanced CKD.^3,13,16–23^
ARNI	CV + Renal	HFrEF.^3,19–22^	Preferred over standard ACEi/ARBs in HFrEF. Associated with better preservation of kidney function.^3,19–22^	Dose reduction is advised if eGFR < 30 mL/min/1,73 m^2^. Mild serum creatinine fluctuations are expected during decongestion.^3^
MRA	CV + Renal	HFrEF; HFmrEF and HFpEF (specifically finerenone); Diabetic CKD with residual albuminuria.^1,3,18,21,22,25,35^	Core pillar of HFrEF therapy. Finerenone added to RAASi/SGLT2i to reduce CKD progression and CV events.^1,3,18,25^	Risk of hyperkalaemia. SGLT2i co- administration is encouraged as it helps lower the risk of hyperkalaemia.^3,13,16–18^

ACEi, angiotensin-converting enzyme inhibitors; ARB, angiotensin II receptor blockers; ARNI, angiotensin receptor–neprilysin inhibitor; ASCVD, atherosclerotic cardiovascular disease; CKD, chronic kidney disease; CKM, cardio-kidney-metabolic; CV, cardiovascular; eGFR, estimated glomerular filtration rate; GLP-1 RA, glucagon-like peptide-1 receptor agonists; HbA1c, haemoglobin A1c; HFpEF, heart failure with preserved ejection fraction; HFrEF, heart failure with reduced ejection fraction; HFmrEF, heart failure with mildly reduced ejection fraction; MRA, mineralocorticoid receptor antagonist; RAASi, renin–angiotensin–aldosterone system inhibitors; SGLT2i, sodium–glucose cotransporter-2 inhibitors; T2DM, type 2 diabetes mellitus.

Of note, in this complex context, general practitioners (GPs) are key actors for detecting early, but frequently asymptomatic CKM indicators. Enabling GPs to initiate SGLT2i requires realistic tools: simplified algorithms, clear referral pathways, and efficient communication channels with specialists.^2,31^ Structured screening visits based on the full screening panel (including eGFR, uACR, HbA1c, lipid profile, BP, ECG, etc.) can allow timely intervention and prevent progression.

Cultural changes in medical training, moving from organ-specific paradigms to a system approach to the disease, are needed. Clinicians should be able to manage overlapping risks and syndromic trajectories from the earliest stages.^31^ This includes addressing the clinical misconceptions and legitimate safety considerations detailed in section ‘Beyond the numbers: clinical and organizational barriers’ to ensure that perceived risks do not lead to unnecessary therapeutic delay. Targeted education and patient-centered communication, particularly around hygiene, hydration, and sick-day management, can further reduce discontinuation rates.

Finally, hospital–territory integration is essential through digital platforms, co-management protocols, and structured referral systems: they may enhance diagnostic efficiency and therapeutic alignment. Defining clear roles, shared tools, and coordinated follow-up is essential to shift from episodic care to a genuinely integrated, risk-based CKM model.

To translate these principles into practice, a pragmatic implementation roadmap may help prioritize actions across healthcare settings. *Table 7* outlines a staged implementation framework for CKM care, including key targets, suggested audit metrics, and responsible stakeholders.

**Table 7:** Prioritized implementation framework for cardiovascular–kidney–metabolic care

Phase and timeline	Strategic priority and target	Suggested audit metrics	Responsible stakeholders
Phase 1: Risk identification (short-term)	Early detection of CKM risk factors before overt disease develops	% of at-risk adults with documented eGFR and uACR. % assessed with SCORE2 calculator	GPs
Phase 2: Targeted initiation (mid-term)	Achieve a 60–70% SGLT2i prescription rate in eligible patients with T2D, HF, or CKD	% of eligible patients and symptomatic HF patients prescribed an SGLT2i	Cardiologists; Diabetologists; Nephrologists
Phase 3: Persistence (long-term)	Ensure long-term adherence and safe management protocols	12-month SGLT2i persistence rate. Frequency of implementation of ‘Sick Day Rules’ in patient records	GPs
Phase 4: Prevention (long-term)	Baseline screening in 100% of eligible population (early CKM stages or asymptomatic metabolic risk factors)	% of the clinic population with a complete annual CKM diagnostic panel.	GPs

CKD, chronic kidney disease; CKM, cardio-kidney-metabolic; eGFR, estimated glomerular filtration rate; GPs, general practitioners; HF, heart failure; SCORE2, Systematic COronary Risk Evaluation 2; SGLT2i, sodium–glucose cotransporter-2 inhibitors; T2D, type 2 diabetes; uACR, urinary albumin-to-creatinine ratio.

## Patient empowerment and communication to support early and sustained sodium–glucose co-transporter 2 inhibitors use

Within an integrated CKM care framework, effective patient communication is central to translating clinical intentions into sustained therapeutic success. As emphasized in the previous sections, early SGLT2i initiation is often hindered by therapeutic inertia and organizational fragmentation, as well as the ambiguous presentation of treatment to patients. Clear, structured, and patient-centered communication markedly enhances risk perception, treatment acceptance, and long-term adherence.

Cardiovascular–kidney–metabolic patients frequently navigate multiple comorbidities and complex treatment regimens, potentially leading to confusion or disengagement. Using plain language and repositioning SGLT2i as organ-protective therapies—rather than solely glucose-lowering agents—helps patients grasp the rationale for early initiation and fosters more meaningful engagement, even during asymptomatic stages.

The value of shared decision-making, particularly in early or asymptomatic CKM stages, must be emphasized. Engaging patients in therapeutic decisions, addressing their concerns, and aligning treatment with patient priorities strengthens the therapeutic alliance and promotes long-term adherence.

## Future perspectives and conclusions

The evolving understanding of CKM syndrome leads to a paradigm shift from reactive disease management to proactive, system-protective strategies. While the benefits of SGLT2i are well established in HF, CKD, and T2D, ongoing trials are now exploring their efficacy in broader populations and earlier disease stages, including non-diabetic patients, those with HF with preserved ejection fraction, and normoalbuminuric CKD, reinforcing the need for an early intervention.

Several key messages emerge from the current evidence and clinical experience. First, early identification of CKM risk and timely initiation of disease-modifying therapies represent a critical opportunity to alter the trajectory of cardiovascular and renal disease. In patients with established high-risk phenotypes, SGLT2i are strongly supported by randomized trials and international guideline recommendations. However, proposals advocating pharmacological intervention at very early stages of CKM, before overt organ damage develops, should be interpreted cautiously. Although biologically plausible and consistent with the disease-modifying mechanisms of SGLT2i, such strategies currently represent an expert extrapolation and remain the subject of ongoing clinical investigation.

Second, improving outcomes across the CKM spectrum requires more than pharmacological innovation. Effective implementation depends on coordinated care models, shared diagnostic frameworks, and clear allocation of responsibilities across specialties, supported by simplified clinical pathways and structured screening strategies.

Ultimately, a truly integrated CKM care model will require close alignment between GPs, specialists, and multidisciplinary teams. The combination of early risk identification, evidence-based therapy, patient engagement, and coordinated care pathways represents the most promising strategy to translate the growing body of CKM evidence into meaningful improvements in real-world clinical outcomes.

## Data Availability

Not applicable.
